# Exploring *Spiroplasma* Biology: Opportunities and Challenges

**DOI:** 10.3389/fmicb.2020.589279

**Published:** 2020-10-21

**Authors:** Shrikant Harne, Pananghat Gayathri, Laure Béven

**Affiliations:** ^1^Indian Institute of Science Education and Research, Pune, India; ^2^INRAE, UMR 1332, Biologie du Fruit et Pathologie, University of Bordeaux, Bordeaux, France

**Keywords:** *Spiroplasma*, shape determination, motility, bacterial cytoskeleton, fibril, MreB, *Spiroplasma* genetics, bacterial division

## Abstract

Spiroplasmas are cell-wall-deficient helical bacteria belonging to the class *Mollicutes*. Their ability to maintain a helical shape in the absence of cell wall and their motility in the absence of external appendages have attracted attention from the scientific community for a long time. In this review we compare and contrast motility, shape determination and cytokinesis mechanisms of *Spiroplasma* with those of other *Mollicutes* and cell-walled bacteria. The current models for rod-shape determination and cytokinesis in cell-walled bacteria propose a prominent role for the cell wall synthesis machinery. These models also involve the cooperation of the actin-like protein MreB and FtsZ, the bacterial homolog of tubulin. However the exact role of the cytoskeletal proteins is still under much debate. *Spiroplasma* possess MreBs, exhibit a rod-shape dependent helical morphology, and divide by an FtsZ-dependent mechanism. Hence, spiroplasmas represent model organisms for deciphering the roles of MreBs and FtsZ in fundamental mechanisms of non-spherical shape determination and cytokinesis in bacteria, in the absence of a cell wall. Identification of components implicated in these processes and deciphering their functions would require genetic experiments. Challenges in genetic manipulations in spiroplasmas are a major bottleneck in understanding their biology. We discuss advancements in genome sequencing, gene editing technologies, super-resolution microscopy and electron cryomicroscopy and tomography, which can be employed for addressing long-standing questions related to *Spiroplasma* biology.

## Introduction

Spiroplasmas are members of the class *Mollicute*s (cell-wall-deficient bacteria) and are characterized by their helical cell shape (Razin et al., 1973). They were discovered as mycoplasma-like helical organisms associated with citrus stubborn and corn stunt diseases (Granados et al., 1968; Laflèche and Bové, 1970; Davis and Worley, 1973). Over the years, many species of *Spiroplasma* have been isolated from a wide variety of hosts, mainly arthropods and plants (Clark et al., 1985; Saillard et al., 1987; Regassa and Gasparich, 2006), and have also been shown to be able to infect humans (Aquilino et al., 2015; Etienne et al., 2018). *Spiroplasma* cells exhibit chemotaxis (Daniels et al., 1980) and move by kinking motility (Shaevitz et al., 2005). The motility characteristics and distinct shape of the *Spiroplasma* cells make it an interesting model system to study the cell-wall independent mechanisms of cell division, shape determination and motility in bacteria.

**FIGURE 1:**
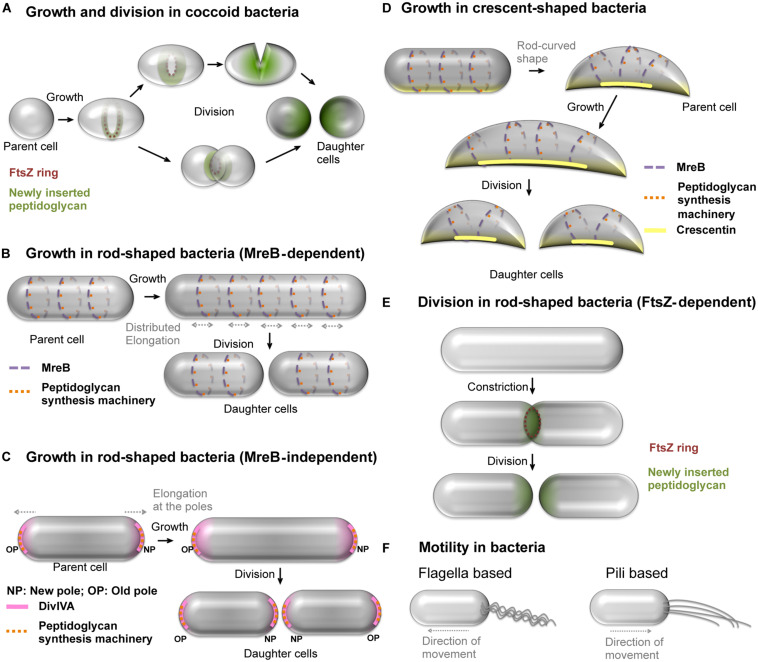
Growth, cell division and motility mechanisms in bacteria. **(A)** Spherical, cell-walled bacteria grow and divide by insertion of peptidoglycan in FtsZ-dependent manner. Alternatively, some spherical bacteria divide by constriction. **(B)** Some of the rod-shaped, cell-walled bacteria such as *E. coli* grow uniformly along the length by uniform insertion of peptidoglycan facilitated by MreB patches and divide to produce daughter cells. The dark blue and light blue colors represent patches of MreB, and associated peptidoglycan synthesis machinery shown as red and pink dots, on the membrane at the front and back of the cell, respectively. **(C)** *M. smegmatis*, a cell-walled bacterium exhibits MreB-independent polar growth by positioning the peptidoglycan synthesis machinery (orange dots) with the help of DivIVA (pink) at the cell poles. **(D)** *Caulobacter crescentus* attains crescent shape by asymmetric growth using crescentin polymers (yellow line) that prevent peptidoglycan insertion at the site of their location. The crescentin polymers are positioned by MreB and the former prevents insertion of new peptidoglycan in its vicinity. The MreB patches present at locations away from crescentin facilitate cell growth, thus leading to crescent shape. **(E)** The most predominant mechanism of cell division by formation and constriction of FtsZ ring at the mid-cell region in cell-walled bacteria. The FtsZ-assisted insertion of peptidoglycan at the mid-cell region results into septum closure and separation of daughter cells. **(F)** Well studied motility mechanisms using appendages in cell-walled bacteria include the (i) flagella dependent swimming and (ii) type IV pili-based twitching motility. See Miyata et al. (2020) for a detailed review on motility mechanisms in bacteria.

Bacterial morphogenesis and motility have attracted considerable attention from researchers for many years. Indeed these processes play a vital role in survival and adaptation to environment, nutrient acquisition, division or host colonization (Yang et al., 2016; Raina et al., 2019). Major advances related to understanding shape determination and cell division have been made using cell-walled bacteria as models. These contain a peptidoglycan cell wall and include cocci (Figure 1A) and rod-shaped bacteria (Figure 1B). In rod-shaped bacteria, MreB and its isoforms (Figure 1B) are essential for guiding peptidoglycan synthesis machinery to distribute growth along the longitudinal surface (Jones et al., 2001; van den Ent et al., 2001; Carballido-López, 2006). Some rod-shaped bacteria without MreB maintain their rod-shape by polar growth (Figure 1C). Further, some bacteria have the ability to deviate from uniform growth of cell wall to attain modification in their rod shape, e.g., the crescent shape in *Caulobacter crescentus* (Figure 1D) and helical shape in *Helicobacter pylori* (Cabeen and Jacobs-wagner, 2005).

In cell-walled bacteria, FtsZ, a tubulin homolog, supports peptidoglycan insertion at the septum during cell division (Figures 1A,E). The positioning of FtsZ in a ring-like arrangement (Z-ring) at the mid-cell region is a crucial step in cell division. The Z-ring formed by multiple overlapping filaments is attached to the cell membrane through adapter proteins such as FtsA and/or ZipA (Hale and De Boer, 1997; Pichoff and Lutkenhaus, 2002). Recent results show that the division of cell-walled bacteria appears to be dependent on the assembly dynamics of FtsZ that guides septum synthesis (Bisson-Filho et al., 2017). However, mechanistic studies carried out on cell-walled bacteria may complicate the decoupling of the respective roles of the peptidoglycan and cytoskeletal proteins in both shape determination and cell division processes.

Spiroplasmas have a helical shape, distinct from those of most *Mollicutes* which are pleomorphic. Spiroplasmas possess the cytoskeletal proteins associated with rod-shape determination and cell division such as MreB, FtsZ, and FtsA. Hence, spiroplasmas serve as a cell-wall-less model system that possess essential cell division and shape determination machinery. However, the paucity of easy genetic modification tools for spiroplasmas has long hampered understanding of the mechanisms of morphogenesis and motility. The advent of genetic modification tools (Renaudin et al., 2014; Terahara et al., 2017), the increase in number of available genome sequences^1^ and improved imaging techniques open up avenues for understanding the molecular basis of morphology and cell division processes in bacteria using the *Spiroplasma* cell as a model system.

In this review, we compare and contrast information available on spiroplasmas with that on mycoplasmas (other *Mollicutes* which naturally lack a cell-wall), and cell-walled bacteria. Based on the comparison we bring out the open questions in understanding motility, shape determination and cell division of *Spiroplasma* cells. Further, we discuss tools and techniques available for understanding *Spiroplasma* biology and strategies for future developments.

## Motility and Cell Division in Genus *Mycoplasma*

### Motility in *Mycoplasma* Species

Motility mechanisms in bacteria typically involve appendages such as flagella and pili (Figure 1F; Miyata et al., 2020). Various motility mechanisms and components exist across species of *Mycoplasma* (Miyata, 2010; Balish, 2014; Garcıa-Morales et al., 2016; Miyata and Hamaguchi, 2016), especially well characterized in *M. mobile*, *M. pneumoniae*, and *M. genitalium*. All described mechanisms involve a multi-protein complex, termed attachment organelle (AO), localized at the cell neck in *M. mobile* (Uenoyama and Miyata, 2005) or at the leading pole in *M. genitalium* and *M. pneumoniae* (Balish et al., 2003; Hasselbring and Krause, 2007). The AO proteins of *M. genitalium* and *M. pneumoniae* are homologous to each other (Hasselbring et al., 2006). However the AO proteins of *M. mobile* and *M. pneumoniae* are not homologous (Balish, 2014), despite the phylogenetic proximity of these two *Mycoplasma* species. A characteristic of bacterial motility is chemotaxis driven by two-component systems. Chemotaxis has been reported in some *Mycoplasma* species (Kirchhoff et al., 1987), but genes encoding putative two-component systems have not been identified in *Mycoplasma* genomes yet.

### Cell Division in *Mycoplasma* Species

Most mycoplasmas are pleiomorphic and do not encode *mreB* genes. Gene *ftsZ* is found in most but not all *Mycoplasma* genomes (Zhao et al., 2004; Lluch-Senar et al., 2010), suggesting that it may not be essential in some mycoplasmas. For instance, upon deletion of *ftsZ* gene, *M. genitalium* divides by a mechanism that involves motility machinery (Lluch-Senar et al., 2010). In *M. pneumoniae*, AO has been shown to be tightly linked to cell division. Partnering of FtsZ and motility machinery might be required for efficient division in motile mycoplasmas harboring an *fts*Z gene (Balish, 2014). According to this model, FtsZ constricts the cell membrane and motility pulls the cells apart.

## *Spiroplasma* Physiology

### Cell Morphology

The elongated morphology of *Spiroplasma* cells ensures a large surface for nutrient import, and helical shape is well adapted for motility in semi-viscous media, a natural environment of spiroplasmas (arthropods’ fluids, plant phloem). Capacity to lose helical morphology appears also essential for some species because, upon entry into host cells, a drastic morphological change into coccoid shapes can occur (Fletcher et al., 1998; Duret et al., 2014).

**FIGURE 2:**
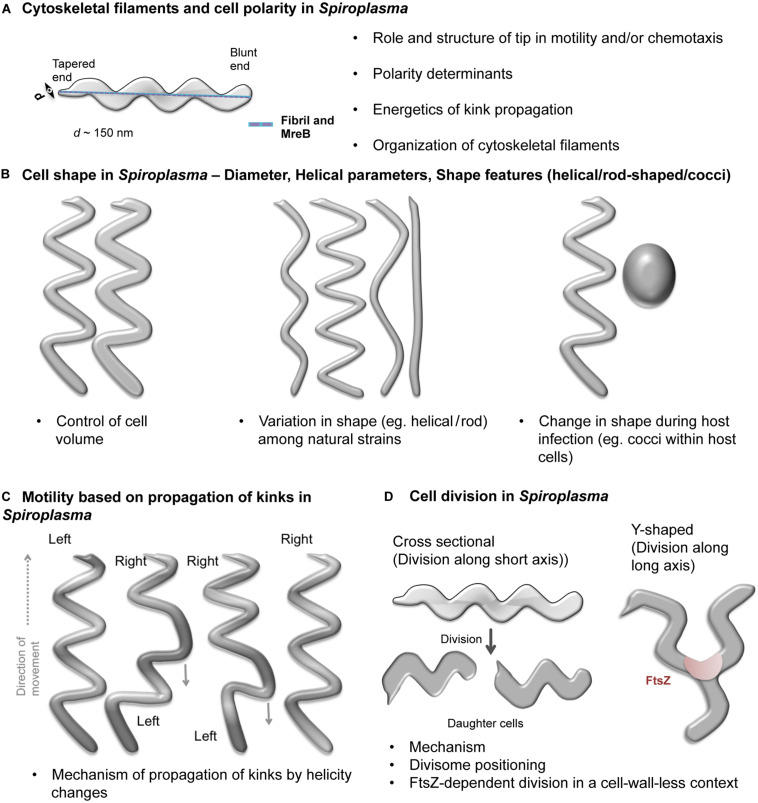
Schematic representations of open questions in *Spiroplasma* biology. Open questions in *Spiroplasma* biology include- **(A)** Organization of cytoskeletal filaments and cell polarity determinants. A *Spiroplasma* cell showing its characteristic helical shape, distinct poles (one is tapered while the other is blunt) and cytoskeletal ribbon (blue) connecting the two poles through the shortest path along the cell body is shown. **(B)** Factors conferring spherical, rod and helical shapes, determinants of helical pitch and cell lengths in *Spiroplasma.* **(C)** Kinking-based motility in *Spiroplasma*. The kink is introduced at one of the poles by change in handedness of the cell and traverses through the cell body. Portions of the cell on either side of the kink have opposite handedness. **(D)** Modes of cell division (cross sectional and Y-shaped) and factors favoring them. The bulleted text in the figures summarizes the open questions.

Fibril (Williamson, 1974), a cytoskeletal protein (Williamson et al., 1991) with a molecular mass of about 59 kilodalton and unique to spiroplasmas is thought to be responsible for helical shape of spiroplasmas (Razin, 1978; Townsend and Archer, 1983). *In vivo*, fibril forms a ribbon of filaments (Charbonneau and Ghiorse, 1984; Schmitt et al., 1984; Townsend and Plaskitt, 1985). A flat ribbon bound at the cytoplasmic side of the cell membrane that spans the entire cell body has been visualized using electron microscopy (Figure 2A; Trachtenberg and Gilad, 2001). The ribbon links the two poles along the shortest helical path. Visualization of *Spiroplasma* cytoskeleton by electron cryotomography (ECT) revealed that filaments of fibril are indeed present close to the cell membrane (Kürner et al., 2005; Trachtenberg et al., 2008). Diffraction analysis of isolated fibrils suggested that conformational switches of the fibril could determine cell shape and helicity by varying the diameter of fibril tetramers in the ribbon (Trachtenberg and Gilad, 2001; Cohen-Krausz et al., 2011). However, this hypothesis will have to be experimentally assessed.

In addition to fibril, MreB filaments were also found to constitute the cytoskeletal ribbon of *Spiroplasma* cells. However, the organization of MreB and fibril filaments within the cytoskeleton is debated (Kürner et al., 2005; Trachtenberg et al., 2008). *Spiroplasma* species possess at least five paralogs of *mreB* gene (Ku et al., 2014). The functional relevance of the different MreB homologs has not yet been proven. However, the presence of 5-8 *mreB* genes in organisms with minimal genome suggests that these proteins may confer a selective advantage to spiroplasmas. In ECT studies on *S. melliferum*, the MreB filaments were identified on the basis of inter-filament spacing (Kürner et al., 2005). This information is insufficient to know if the filaments were made of one or more paralogs of MreB. It is unknown if one or more MreB paralogs act in association with fibril in *Spiroplasma* morphogenesis and if the MreBs perform overlapping functions (Harne et al., 2020).

Besides helicity, *Spiroplasma* cells also show polarity, with a tapered end (tip structure) associated to an electron dense core and a blunt or rounded end (Figure 2A; Garnier et al., 1981; Ammar et al., 2004). The tip structure seems to expose the adhesins required for attachment to cells of the vector insect for the plant pathogenic *Spiroplasma* species (Ammar et al., 2004; Duret et al., 2014). Another interesting feature of spiroplasmas is their diameter of about 100–150 nm, much smaller than a typical bacterial cell (Figures 2A,B). The factors determining the diameter of the cell are currently unknown.

### Motility

During motility, *Spiroplasma* cells exhibit twitching, bending and flexing and rotation along the helical axis of the cell (Gilad et al., 2003). Spiroplasmas swim by formation and propagation of kinks (Figure 2C). Cell helicity seems to facilitate processive movement of kinks along the cell body (Shaevitz et al., 2005). Cells having lost helicity upon decrease of the transmembrane electric potential become non-motile (Béven and Wróblewski, 1997). The helical, non-motile mutant of *S. citri* obtained by Jacob et al. (1997) indicates that in addition to helical shape a functional *scm*1 gene is required for rotation about the helix axis. However the exact role of Scm1 in motility of spiroplasmas remains to be elucidated.

Fibril filaments are proposed to be responsible for *Spiroplasma* motility in addition to conferring helical shape (Razin, 1978). The *Spiroplasma* fibrils are distinct from the flagellar machinery; these filaments are intra-cellular. While fibril filaments are believed to undergo co-ordinated conformational changes to produce kinks in the cell body during motility, the role of MreBs in *Spiroplasma* motility is unknown. The introduction and propagation of kinks requires a flexible cell body. The absence of cell wall in spiroplasmas probably provides flexibility for enabling “kinking motility.”

High resolution video microscopy and electron microscopy studies suggested that the kinks are always introduced at the tapered end (Shaevitz et al., 2005; Liu et al., 2017). It was later shown that the kinks are preferentially initiated at the tip, but initiation can occasionally occur at the opposite pole and propagate along the whole cell body (Boudet et al., 2018). In *S. eriocheiris*, chemotactic activity was clearly shown to be linked to change in cell helicity and reversal of motion (Liu et al., 2017). In the absence of homologs of conventional chemotaxis proteins found in other bacteria, it is possible that the tip structure is involved in the change in helicity from front to back (Liu et al., 2017). For some *Spiroplasma* species, attachment to host cells occurs through the tip (Ammar et al., 2004; Duret et al., 2014) and involves binding to glycoconjugates (Duret et al., 2014). In some *Mycoplasma* species, the attachment organelle also binds glycosylated compounds (Kasai et al., 2015). It is thus tempting to propose roles for *Spiroplasma* tip in motility similar to those of AO in *Mycoplasma* species (Liu et al., 2017). Since different mechanisms exist in *Mycoplasma* genus, it is quite possible that another independent mechanism evolved in *Spiroplasma*.

### Cell Division

Most of the *Mollicutes* have only 4 (*mraZ*, *mraW*, *ftsA*, and *ftsZ)* of over 15 genes coded by the division and cell wall (*dcw*) cluster in cell-walled bacteria (Zhao et al., 2004). Given the reduced genomes of these bacteria, this suggests that these four proteins constitute the machinery sufficient for cell division in bacteria lacking a cell wall. However, it is unknown if all these proteins are necessary for *Spiroplasma* cell division.

Two modes of cell division have been proposed for *Spiroplasma* (Figure 2D). The first mode was based on observation of *S. citri* growth. It proposes that *Spiroplasma* cells grow at one of the two poles by insertion of new membrane components and divide by one or multiple constrictions along the short axis of the cell (Garnier et al., 1981, 1984). In contrast, in *S. poulsonii*, another mechanism involves longitudinal fission (Y-shape) dependent on FtsZ (Ramond et al., 2016). It is unclear if both these mechanisms exist in all *Spiroplasma* species and if the choice between them depends on specific circumstances or environmental conditions. If Y-shaped division was the predominant mechanism, the *Spiroplasma* cells should have a fixed length. However, cells of varying lengths are always observed in growing cultures. This suggests that the Y-shaped division may be an alternate mode of cytokinesis occurring more or less often depending on the species. The constituent proteins of the *Spiroplasma* cell division machinery and mechanism governing their positioning at the appropriate site needs to be investigated.

Figure 2 summarizes the open questions in *Spiroplasma* biology. How they can be addressed using a combination of approaches is discussed below.

## Available Tools and Future Directions

### Morphology Variants, Deletion Mutants and Genome Analysis

Some of the attempts to understand gene function in *Spiroplasma* have been conducted through random mutagenesis using ultraviolet (UV) light (Labarère and Barroso, 1989) or by insertion of transposon (Foissac et al., 1997). These methods require extensive functional or morphological screening to identify mutants of interest. A major hurdle in preparing *Spiroplasma* mutants is the low recombination rate. Disruption of multiple genes simultaneously is technically very challenging (Duret et al., 2005). The limited number of antibiotics to which *Spiroplasma* is sensitive also makes the process difficult (Duret et al., 2005). In general, fundamental cellular processes in *Spiroplasma* remain poorly understood due to the above difficulties associated with genome modifications.

Nonetheless, the toolbox for genetic manipulation is continuously expanding (Renaudin et al., 2014). Moreover, the possibility to insert and modify a *Mycoplasma* genome in yeast and transplant it back to a recipient cell gives hope that this technology will soon be applicable to spiroplasmas. An alternate approach could be to perform vector-based expression of recombination proteins as carried out in *M. hyorhinis* (Ishag et al., 2017). One may also explore the possibility of standardizing CRISPR-Cas (Clustered Regularly Interspaced Short Palindromic Repeats and CRISPR associated genes) for specific gene targeting (Ipoutcha et al., 2019) in *Spiroplasma*. Given the uncertainties and inefficiency of currently available recombination tools in *Spiroplasma*, use of heterologous potent recombination machinery may facilitate its target gene disruption efficaciously.

Given the challenges in genetic manipulation, one may exploit the *Spiroplasma* species differing in their helicity (Figure 2B; e.g., cells of *S. platyhelix* are flattened, helical filaments while *S. ixodetis* cells are tightly helical) to understand helicity determinants or explore the naturally occurring mutants (e.g., *S. citri* ASP-I). The molecular basis for helicity can be studied by performing genome analysis and checking expression levels of cytoskeletal proteins. Indeed, natural species differ in their number of MreB paralogs, in cell diameter and helix pitch (Figure 2B; Regassa and Gasparich, 2006). These studies are feasible given the increasing number of *Spiroplasma* genomes available (NCBI database for genome assemblies contains 31 *Spiroplasma* genomes out of which 22 were deposited during the five last years). Naturally occurring strains of *Spiroplasma* with morphology variations have been isolated [e.g., *Spiroplasma citri* ASP-I (Townsend et al., 1977)], which can be characterized for identifying genetic defects (Harne et al., 2020). For example, strains which exhibit helical morphology (Abalain-Colloc et al., 1987; Whitcomb et al., 1997) despite a probable disruption (a stop codon) in *fibril* gene such as *S. sabaudiense* Ar-1343 (Chang T. H. et al., 2014) and *S. helicoides* TABS-2 (Shen et al., 2016) could provide further insights into the functionality of the *fibril* gene.

### Imaging Technologies

Dark-field light microscopy is commonly used in order to visualize unlabeled, living spiroplasmas in liquid. Motility of single cells and kink propagation can be quantified using this technique (Boudet et al., 2018). Recently, the development of a line-scanning optical trap was applied to record shape deformation of the *Spiroplasma* body under various stress conditions (Koch and Rohrbach, 2012, 2014). For live cell imaging, it is essential to create gene disruption strains and complementation of the gene with a fluorescently tagged version of the protein encoded by the gene. These can provide clues about cell division process and role of cytoskeletal proteins in motility. Successful visualization of YFP-tagged protein in *S. eriocheiris* cells in the recent past (Terahara et al., 2017) is a promising step toward identifying intra-cellular localization of proteins. However, considering the diffraction limit of light and the small diameter of the cell (∼0.15 μm), super resolution microscopy techniques are essential for *Spiroplasma* imaging experiments.

In contrast, the small diameter of the cell (∼100–150 nm) makes *Spiroplasma* an ideal system to explore using electron cryotomography (ECT). The technique is useful for three-dimensional (3D) visualization of samples within the cell in a near-native state, and is commonly used for visualization of protein filaments, secretion systems, chemoreceptor arrays and localization of proteins as a part of protein assembly (Briegel et al., 2006, 2012; Basler et al., 2012; Chang Y. W. et al., 2014). It has also been applied to *Spiroplasma* to visualize arrangement of protein filaments in cytoskeleton (Kürner et al., 2005). In this context, conjugating fluorescence and electron microscopy using cryo-correlative light and electron microscopy (CLEM) should prove useful for visualization of intra-cellular localization of protein with high precision in a near-native state. Comparative CLEM studies can be performed on wild type cells, (naturally occurring or artificially generated) mutants and rescued mutants of *Spiroplasma* to identify localization of cytoskeletal proteins such as fibril, MreB, FtsZ.

## Conclusion

*Spiroplasma* is a unique bacterium without a cell wall, which possesses a definite helical shape and is motile. Despite the absence of a cell wall, it utilizes the cytoskeletal elements such as MreB and FtsZ, like cell-walled bacteria, for physiological processes such as cell elongation, cell division and motility. For understanding the molecular mechanisms of these processes in biology, *Spiroplasma* thus qualifies to be a promising model organism.

## Author Contributions

All authors contributed to the drafting and revising process and approved the final version of this manuscript.

## Conflict of Interest

The authors declare that the research was conducted in the absence of any commercial or financial relationships that could be construed as a potential conflict of interest.

## Footnotes

1 https://www.ncbi.nlm.nih.gov/genome/?term=spiroplasma
